# Meniscal tear as potential steering factor for inflammation may aggravate arthritis: two case reports

**DOI:** 10.1186/1752-1947-8-137

**Published:** 2014-05-06

**Authors:** Priya Kulkarni, Soumya Koppikar, Shantanu Deshpande, Narendrakumar Wagh, Abhay Harsulkar

**Affiliations:** 1 Interactive Research School for Health Affairs (IRSHA), Bharati Vidyapeeth University, Medical College Campus, Pune-Satara Road, Pune 411043, India; 2 Department of Orthopaedics, Bharati Hospital, Pune-Satara Road, Pune 411043, India

**Keywords:** Arthritis, Cartilage degeneration, GAG, IL-1β, Inflammation, Meniscal tear, Nitric oxide, Synovial fluid

## Abstract

**Introduction:**

Meniscal tear is thought to play a crucial role in onset as well as progression of arthritis. However, role of cytokine response to meniscal injury and resulting inflammation is not clearly understood. Because synovial fluid is juxtaposed to cartilage and serves as a biological connection between chondrocytes and synoviocytes, we chose synovial fluid analysis to ascertain biochemical response associated with a meniscal tear.

**Case presentation:**

We report the cases of two patients with clinically different inflammatory arthritis, both of whom are Indian men. Patient 1 was 30 years of age, and patient 2 was 50 years of age. They both had a history of meniscal tear, which we confirmed by magnetic resonance imaging scans. Synovial fluid samples obtained from these two patients were analyzed for proinflammatory markers, such as interleukin 1β (IL-1β) and nitric oxide, and also for glycosaminoglycan as a cartilage degradation indicator. Relatively high levels of IL-1β (2000.0±15.7pg/ml) and nitric oxide (4.73±0.05μM/ml) and relatively low glycosaminoglycan (93.75±6.3μg/ml) were observed in patient 1, corroborating the diagnosis of traumatic meniscal tear. Compared to patient 1, Patient 2 had relatively low levels of IL-1β (54.55±14.5pg/ml) and nitric oxide (20.00±0.6μM/ml) and remarkably high glycosaminoglycan levels (553.33±1.7μg/ml), coupled with significant osteophytes and profound cartilage loss, which indicated severe arthritis and a diagnosis of degenerative meniscal tear.

**Conclusion:**

The elevated levels of inflammatory IL-1β aggravated the severity of arthritis attributable to meniscal tear in both patients, as found in follow-up visits. This was quite evident in patient 2, whereas patient 1, being younger, had less serious symptoms. Meniscal tear has emerged as a potential confounding factor in arthritis with different clinical backgrounds, which leads to increased levels of inflammation and results in accelerated disease progression.

## Introduction

The knee joint meniscus is fibrocartilaginous in nature and designed to provide joint stability, load distribution, shock absorption and joint lubrication [1]. Meniscal damage is common in young, active individuals as well as in older people with degenerative conditions. Although patients with meniscal damage may remain asymptomatic for a long time, it is understood that meniscal tears (MTs), even due to minor trauma, have the potential to lead to an insidious onset of arthritis [1].

Early diagnosis of arthritis remains a challenge, and inflammatory arthritis (IA) is associated with high and progressive patient morbidity. According to current understanding, the pathogenesis of IA revolves around inflammation in the synovium, which is regulated by interplay of pro- and anti-inflammatory cytokines. The imbalance between these proinflammatory and anti-inflammatory agents favors chronic inflammation, which leads to cartilage destruction. Although, the interplay of various cytokines is unclear in this context, interleukin 1β (IL-1β), a major proinflammatory cytokine known to be involved in the pathogenesis of arthritis, is the focus of our present case report.

Synovial fluid (SF) analysis is thought to be potentially helpful in understanding the progression of arthritis because it bridges a biochemical communication between chondrocytes and synoviocytes [2]. Cartilage damage and inflammatory changes begin at early stage of the disease, thus it could be informative to analyze SF at both early and advanced stages. In our present report, we describe the cases of two patients with IA (Kellgren-Lawrence (KL) scale grade 1 and grade 3, respectively) having common history of MT. IL-1β, the major proinflammatory cytokine, is known to be involved in IA pathogenesis, so we measured it along with nitric oxide (NO), nitrate/nitrite and glycosaminoglycan (GAG) in SF samples collected from the patients.

The protocol for the treatment of our patients was approved by our Institutional Ethical Committee, constituted for this purpose (BVDU/MC/56). The SF (1:100 dilutions) was further checked for NO assay and nitrate/nitrite, GAG and IL-1β levels evaluation.

## Case presentation

### Patient 1

Patient 1 was a 30-year-old Indian man who presented to our hospital with complaints about right knee, right elbow and right ankle joint pain of 18 months’ duration. He reported interrupted joint swelling. Large synovial effusion was noted on his left knee. He had no history of any major physical, mental or surgical illness. He denied use of alcohol or tobacco on a regular basis. His physical examination revealed swelling, and tenderness on his left knee. Laboratory investigation results showed that human leukocyte antigen B27 (HLA-B27) was positive, but rheumatoid factor (RF), anticyclic citrullinated peptide (anti-CCP) antibodies and antinuclear antibodies (ANAs) were negative. The radiological findings for both the patient’s knees (anteroposterior view) were normal, but magnetic resonance imaging (MRI) of his left knee joint showed a partial tear of the anterior cruciate ligament (ACL) and the posterior horn of the medial meniscus. The patient is a bus driver by profession and thus is prone to traumatic knee injury. Intraoperative arthroscopy of his knee showed minor nonspecific changes with articular cartilage softening. Hence, we assigned a grade of 1 on the KL scale. The patient has not reported any worsening of symptoms and is managing to carry out his daily activities, as he informed us in follow-up visits.

### Patient 2

Patient 2 was a 50-year-old Indian man who presented to our hospital with left knee joint locking and pain of more than 6 months’ duration. He reported a history of positive RF in his early years, although he had no specific physical or surgical illness. He did not report any regular medication or alcohol consumption. Left knee radiographs (lateral and anteroposterior views) showed reduction of the medial joint space and osteophyte formation at the medial and lateral joint lines. A start of posterior osteophyte formation was also observed, thus indicative of HL grade 3 IA. The patient’s MRI scan revealed a left to mid-meniscus tear, which was confirmed by diagnostic arthroscopy. A partial meniscectomy was performed at the time of arthroscopy. In his recent follow-up visits, the patient has reported aggravated left knee joint pain and swelling along with phalangeal pain in both hands.

### Surgical procedures

To obtain SF from patient 1, arthrocentesis was performed by an experienced surgeon. Aspiration of SF was carried out with strict aseptic precautions. The left knee was cleaned and draped, and a sterile 18-gauge needle connected to a 10ml sterile syringe was used to aspirate the knee. As this procedure itself is done by a single needle prick, local anesthesia was not used. It also avoids contamination of the SF. A two-step procedure was used to carry out arthrocentesis. The first step was to puncture the skin, and the second was a puncture of the synovial capsule. SF was collected from patient 2 in the same standard way, followed by arthroscopy.

A Griess reaction was used for estimation of NO levels in the SF samples [3]. The collected frozen SF samples were taken in triplicates and diluted appropriately by adding 1% sulfanilic acid and 0.1% *N*-(1-naphthyl)ethylenediamine dihydrochloride prepared in 5% phosphoric acid in a 1:1 ratio. After incubation at room temperature for 10 minutes, the absorbance was measured at 540nm in a spectrophotometer (Bio-Rad Laboratories, Hercules, CA, USA). Calculations on a standard curve were obtained by increasing concentrations of sodium nitrate.

The activities of nitrate/nitrite in SF samples were determined by using a kit (Cayman Chemical, Ann Arbor, MI, USA) in a plate format according to the manufacturer’s instructions. Optical density was measured at 540nm using a microplate reader (Bio-Rad Laboratories).

A 1,9-dimethyldimethylene blue-based dye binding assay was performed for measurement of GAG in SF samples. Chondroitin sulfate was used as a standard [3]. The GAG levels were expressed as microgram equivalents of chondroitin sulfate per milliliter of SF. IL-1β was measured in all SF samples using an enzyme-linked immunosorbent assay kit (Abnova, Walnut, CA, USA) in a plate format according to the manufacturer’s instructions provided in a kit manual.

## Discussion

It is well-understood that knee joint menisci transmit 45% to 60% of joint load to anywhere in the body. This function is supported largely by the microstructure of meniscal cartilage, which is composed of circumferentially oriented collagen fibers woven together with radial fibers. This structure looks like tension rods that maintain their shape and structure when axially loaded. Meniscal injury induces intermediate inflammatory changes in the synovium, which may lead to mild to moderate synovitis [4] and to subluxation and ultimately maceration. MT also appears to be a strong predisposing factor for progression of preexisting arthritis [5]. However, little is known regarding the influence of MT on the biochemical milieu of SF. In particular, it is not very clear how the menisci are responsible for producing mediators of inflammation that accelerate to the pathogenesis of arthritis.

The two patients described herein were diagnosed with IA with different etiologies. Patient 1 is a 30-year-old man with KL grade 1 arthritis of the knee with relatively high IL-1β levels (2000.0±15.7pg/ml). Patient 2, a 50-year-old man, had comparatively lower levels of IL-1β (54.55±14.5pg/ml). It is well-understood that IL-1β is a potent biomarker for tissue inflammation. Unlike the significant differences in their IL-1β levels, both patients had matching nitrate/nitrite and NO levels (Table 1).

**Table 1:** **Proinflammatory biomarker levels in synovial fluid obtained from two arthritis patients**^**a**^

**Patient**	**IL-1β levels (pg/ml)**	**NO levels (μM/ml)**	**Nitrate/nitrite levels (μM)**	**GAG levels (μg/ml)**	**KL scale grade**
1	2000.0±15.7	4.73±0.05	20.94±0.4	93.75±6.3	1
2	54.55±14.5	20.00±0.6	25.5±0.7	553.3±1.7	3

^a^GAG, Glycosaminoglycan; IL-1β, Interleukin 1β; KL, Kellgren-Lawrence; NO, Nitric oxide.

In patient 1, clinical signs and symptoms such as pain, restricted left knee joint movement, swelling, tenderness and joint effusion were suggestive of MT, which was confirmed by MRI, a reliable diagnostic tool. The presence of SF effusion on an MRI scan is a typical indication of MT (both ACL and posterior horn medial MT) [6]. The onset of the symptoms in patient 1 was not acute. The pathological investigations showed positive HLA-B27 and negative RF, anti-CCP antibodies and ANAs, which were indicative of inflammation following traumatic MT. Because patient 1 is a bus driver by profession, he is prone to meniscal damage. Spector *et al.*[7] reported that the prevalence of knee symptoms in the driving profession is estimated to be between 10% and 50%. Truck driving has been noted to be one of the top two occupations with regard to knee problems causing compensable work-related musculoskeletal disorder (WMSD) state fund claims. Sprain and meniscal/ligament disruptions are the leading diagnoses among the groups for knee-related WMSD claims. In patient 1, the MT was traumatic, explaining his high IL-1β level.

The MT in patient 2 was perhaps of a more degenerative nature and a result of a long history of arthritis. Cartilage loss at an early age and degenerative MT were strongly supported by his high GAG elevation and the presence of osteophytes. In addition, high NO levels, together with elevated nitrate/nitrite levels, in patient 2 may indicate stress and inflammation. Degenerative MT and partial meniscectomy performed during arthroscopy in patient 2 could also be responsible for KL grade 3 radiographic findings (joint space reduction and osteophyte formation), thus aggravating his preexisting IA. During recent follow-up visits, patient 2 has reported increased left knee joint pain, indicating rapid wearing down of cartilage along with phalangeal pain in both hands, and severe backache, suggesting deteriorating disease condition.

Englund *et al*. 2001 [8] demonstrated that degenerative MT was thought not only to be the first sign of but also the accelerated development of arthritis, which is often associated with increased cartilage damage [9]. Earlier, Rand [10] observed denudation of the medial meniscus as the most commonly involved degenerative change. Interestingly, in patient 2, the affected degenerative meniscus was of that type. Anderson *et al*. [11] reported alterations in different proteins, including proinflammatory cytokines, following joint injuries, which are attributed to joint degeneration. In humans, however, no specific data are available on perturbations in SF following MT. It is known that release of cytokines such as IL-1β and tumor necrosis factor α is the initial response to joint injury following tissue inflammation. IL-1 receptor antagonist (IL-1Ra) together with mediated inhibition of IL-1β is therefore an attractive treatment strategy that has been proved to be effective therapeutically in animal models [12,13]. Study of IL-1Ra response in humans could be an important clue to management that promotes meniscal repair [11] and decelerates cartilage degeneration.

## Conclusions

On the basis of this report of two cases, it is apparent that MT serves as a confounding factor of considerable importance for cartilage degeneration, even with different pathophysiologies. MT is known to trigger inflammation through increased IL-1β levels. McNulty *et al.*[14] demonstrated that IL-1β can potentially interfere with meniscal repair through upregulation of matrix metalloproteinases (MMPs). However, we did not measure our patients’ MMP levels. The high IL-1β levels in both patients was likely to have inhibited integrative MT repair and might have led to further cartilage loss, as was evident in patient 2. Patient 1, being younger than patient 2, has a better chance for recovery because the success of MT repair is age-dependent [15]. The prudent clinical advice generated by this case report for the treatment of patients with different types of arthritis is to avoid injury to prevent acceleration of disease progression.

## Consent

Written informed consent was obtained from the patients for publication of this case report and any accompanying images. Copies of the written consents are available for review by the Editor-in-Chief of this journal.

## Abbreviations

GAG: Glycosaminoglycan; IA: Inflammatory arthritis; IL-1β: Interleukin-1β; IL-1Ra: Interleukin-1Receptor antagonist; KL: Kellgren lawrence grading; MRI: Magnetic resonance imaging; MT: Meniscal tear; NO: Nitric oxide; RA: Rheumatic arthritis; SF: Synovial fluid.

## Competing interests

The authors declare that they have no competing interests.

## Authors’ contributions

PK and AH initiated the work and prepared the manuscript. SK was responsible for the analysis. SD and NW are orthopedic surgeons who diagnosed the patients, and they collected the samples and contributed to the clinical care of the patients. PK was responsible for the logistics and collection of clinical data. All the authors read and approved the final draft of the manuscript for publication.
